# Supplementary material for: The genome sequence of the Marsh Click-beetle, Actenicerus siaelandicus (Müller, O.F., 1764)
Source: Wellcome Open Res. 2025 Feb 19;10:81. [Version 1] doi: 10.12688/wellcomeopenres.23719.1 (PMC11880762; doi:10.12688/wellcomeopenres.23719.1)
Supplement: Peer review history

# Reviewer report: Reviewer response for version 1

**DOI:** 10.21956/wellcomeopenres.26167.r119880

**Published:** 2025-03-04

**Authors:** Hume B Douglas

**Affiliations:** 1 Agriculture and Agri-Food Canada, Ottawa, Canada

Well written. I suggest a few minor wording changes only:

 I suggested adding the subfamily to the title, and removing the word 'Prosterninae'

 Maybe specify 'acidic' wetland habitats.

 Please replace 'pectinate' with serrate

 Please define 'scrub'

 This will be a good addition to elaterid systematics. We also submitted a specimen of Beckerus apressus for genome sequencing this year.

 Thanks,

 Hume Douglas

Are sufficient details of methods and materials provided to allow replication by others?

Yes

Is the rationale for creating the dataset(s) clearly described?

Yes

Are the datasets clearly presented in a useable and accessible format?

Yes

Are the protocols appropriate and is the work technically sound?

Yes

Reviewer Expertise:

Beetle taxonomy

I confirm that I have read this submission and believe that I have an appropriate level of expertise to confirm that it is of an acceptable scientific standard.

# Reviewer report: Reviewer response for version 1

**DOI:** 10.21956/wellcomeopenres.26167.r119216

**Published:** 2025-03-01

**Authors:** Ruiqi Li

**Affiliations:** 1 University of Colorado Boulder, Boulder, Colorado, USA

Sivell et al. presents a high-quality genome of the Marsh Click-beetle, *Actenicerus siaelandicus. *The methods is rigorous and the assembly seem to be high-quality. I only have a few minor suggestions:

 1. In the introduction, it would be great if the authors could write a few sentences on what biological questions the genome could help us answer. 

 2. Is the chromosome number consistent with other species in the same genus/family? Consider doing a synteny analysis with a few closely related species

Are sufficient details of methods and materials provided to allow replication by others?

Yes

Is the rationale for creating the dataset(s) clearly described?

Yes

Are the datasets clearly presented in a useable and accessible format?

Yes

Are the protocols appropriate and is the work technically sound?

Yes

Reviewer Expertise:

Genomics

I confirm that I have read this submission and believe that I have an appropriate level of expertise to confirm that it is of an acceptable scientific standard.

## References

[1] : Photosymbiosis shaped animal genome architecture and gene evolution as revealed in giant clams. *Commun Biol* .2025;8(1) : 10.1038/s42003-024-07423-8 7 10.1038/s42003-024-07423-8 39755777 PMC11700175
